# Multicentric Reticulohistiocytosis Presenting As Acute Pericarditis With Moderate-Sized Pericardial Effusion: A Case Report and Review of Multicentric Reticulohistiocytosis Treatment

**DOI:** 10.7759/cureus.39953

**Published:** 2023-06-04

**Authors:** Saikiran Mandyam, Jayabharath Onteddu, Rubela Ray, Rafaela Basso, Fadi Bader, Nirmal K Onteddu

**Affiliations:** 1 Internal Medicine, Southeast Health Medical Center, Dothan, USA; 2 Internal Medicine, Viswabharathi Medical College, Kurnool, IND; 3 Internal Medicine, Bankura Sammilani Medical College, Bankura, IND; 4 Internal Medicine, Flowers Hospital, Dothan, USA

**Keywords:** immunomodulators, steroids, methotrexate, pericarditis, pericardiac effusion, multicentric reticulohistiocytosis

## Abstract

Multicentric reticulohistiocytosis (MRH) is a rare, class IIb non-Langerhans cell histiocytosis associated with skin and joint involvement. It is more prevalent (80%) in Caucasian females in their fifth to sixth decade of life. Patients usually demonstrate symptoms and signs of symmetric polyarthritis and papulonodular cutaneous lesions. In addition to skin and joints, multiple organs can be involved, such as the lung (pleural effusion, interstitial fibrosis, hilar lymphadenopathy), heart (pericardial effusion, myocarditis), gastrointestinal system, and urogenital system (genital tract and kidney). Pericardial involvement is a rare manifestation, and around three cases have been reported in the literature so far. Our case report is a valuable contribution to the literature, which aids clinicians in contemplating MRH as one of the differentials among patients presenting with pericardial effusion. We described the characteristics of MRH along with its differentiating features from other autoimmune conditions and management.

## Introduction

Multicentric reticulohistiocytosis (MRH) is a rare, class IIb non-Langerhans cell histiocytosis associated with skin and joint involvement. It is more prevalent (80%) in Caucasian females in their fifth to sixth decade of life [1,2]. Male to female ratio of the MRH prevalence is 1:3 [3,4]. Skin-colored papulonodular lesions are typically seen all over the body. Pathognomonic lesions are usually observed around the periungual region. These lesions often coalesce, giving a string of pearls or coral bead appearance. The joint involvement in MRH is quite destructive, and involvement of distal interphalangeal joints is typically seen in more than 75% of patients, which can be used as a distinguishing feature to differentiate from other erosive arthritis, such as rheumatoid arthritis [5]. In addition to skin and joints, multiple organs can be involved, such as the lung (pleural effusion, interstitial fibrosis, hilar lymphadenopathy), heart (pericardial effusion, myocarditis), gastrointestinal system, and urogenital system (genital tract and kidney) [1]. To our best knowledge, around 300 cases of MRH have been reported in the literature [4]. Here we present a case report of a patient with biopsy-proven MRH who presented with pericardial involvement, a very rare manifestation.

## Case presentation

A 34-year-old African American male with a past medical history of biopsy-proven MRH was admitted to our facility with chest discomfort, worse in the supine position and relieved by leaning forward. The patient also endorsed shortness of breath over a few days prior to the presentation. 

**Figure 1:**
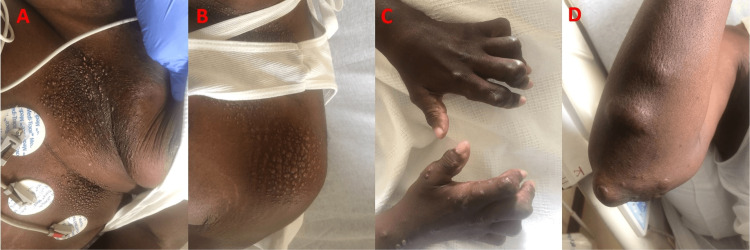
Papulonodular cutaneous lesions involving left chest, left shoulder, bilateral fingers, and right elbow.

**Figure 2:**
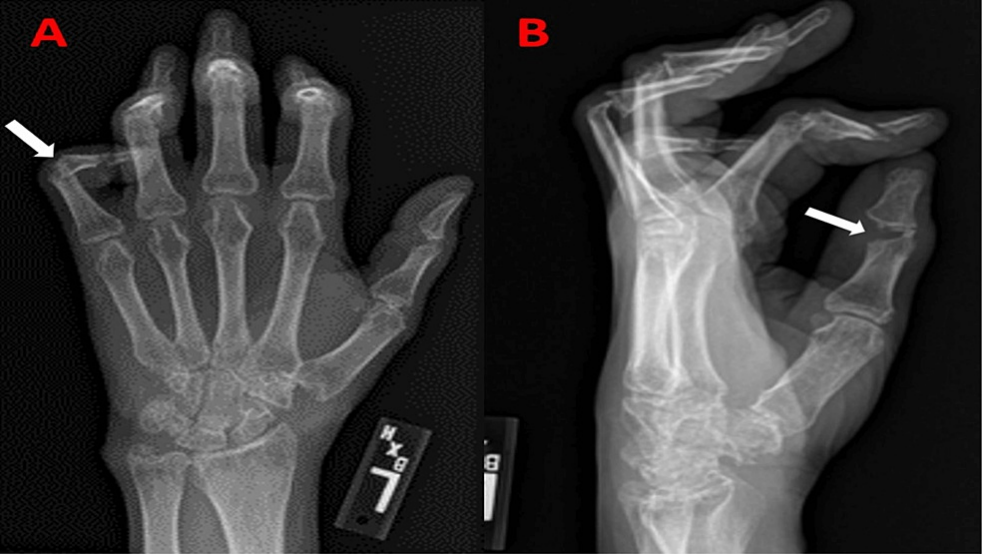
X-ray left hand posteroanterior (A) and lateral view (B) demonstrating destructive interphalangeal joints (white arrows) with loss of bony substance around the interphalangeal joint.

**Figure 3:**
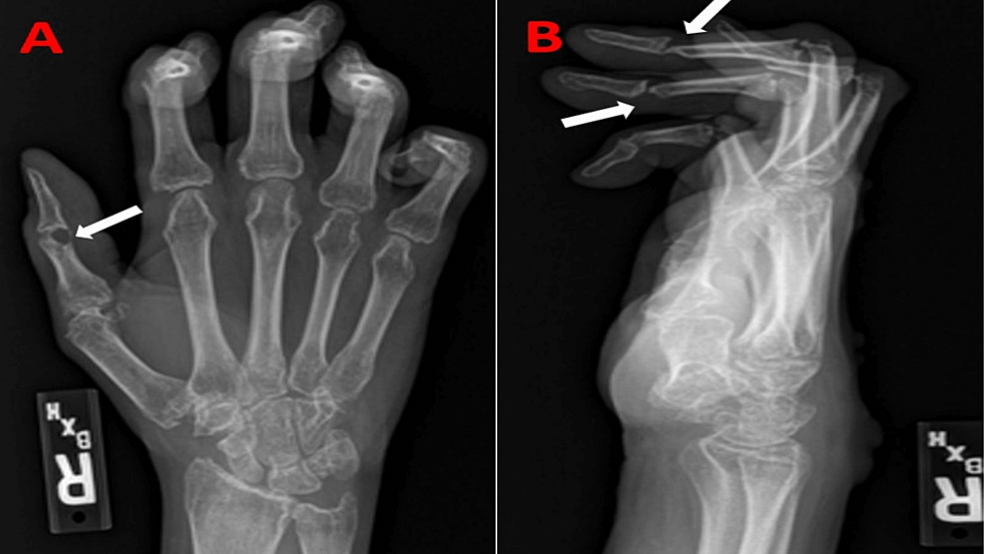
X-ray right hand posteroanterior (A) and lateral (B) views demonstrating severe bone destruction around the interphalangeal joints (white arrows).

**Figure 4:**
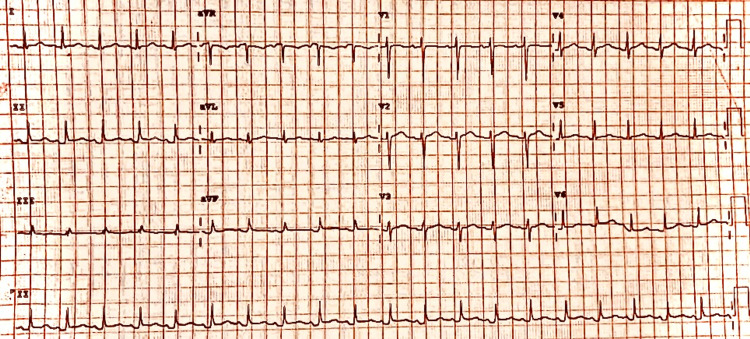
EKG demonstrating PR depressions in multiple leads, ST elevations in inferior leads. Note the low voltage QRS complexes with variable QRS amplitudes suggestive of electrical alternans due to pericardial effusion.

**Figure 5:**
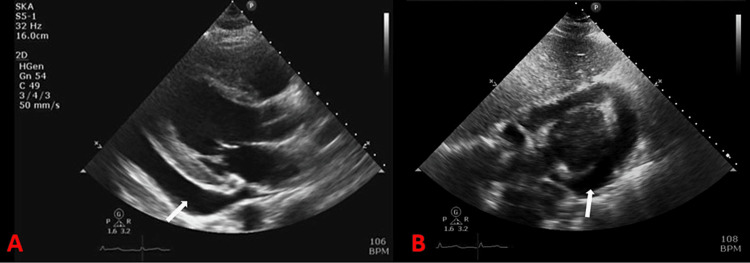
Transthoracic echocardiogram - parasternal long-axis (A) and subcostal (B) views demonstrating large pericardial effusion (white arrows).

He has had intermittent flare-ups of arthritis that did not completely resolve with prednisone and methotrexate and subsequently he was maintained on adalimumab. Upon physical exam, patient was tachypneic and tachycardic with muffled heart sounds and no appreciable pericardial rub. Pulsus paradoxus was not elicited. Upon further examination, the patient had multiple large, skin-colored subcutaneous nodules over the ventral surface of the forearms (Figure 1). Several smooth, well-defined, firm, reddish brown papules were also noted on the skin, more specifically over the dorsal surface of the trunk (Figure 1). There was also coral beading around the cuticle area of the fingers. Interphalangeal joints were significantly distorted, as shown in the X-ray of the hand (Figures 2, 3). Electrocardiogram demonstrated sinus tachycardia, with diffuse PR depressions and ST elevations in certain leads raising suspicion for acute pericarditis (Figure 4). Transthoracic echocardiogram demonstrated normal left ventricular ejection fraction with moderate to large pericardial effusion predominantly posterior, with no signs of tamponade (Figure 5). The patient denied weight loss, recent upper respiratory tract infections, or skin rashes. No evidence of pneumonia or mediastinal lymph node enlargement in the computed tomography of chest with contrast. Pneumonia panel, coronavirus disease 2019 (COVID-19) polymerase chain reaction (PCR), Legionella, and Streptococcus pneumoniae antigen testing were negative. Autoimmune disease workup is usually not recommended for pericarditis if there is no clinical suspicion. However, the patient had a history of MRH, and hence he had already undergone outpatient work-up prior to presenting to the hospital, which included anti-Ro, anti-La anti-Smith antibody, anti-ribonucleopeptide antibody, anti-scl-70 antibody, anti-Jo-1 antibody, antinuclear antibody, anticardiolipin antibody, and Beta 2 glycoprotein antibodies, which were all undetectable. One of the Lupus anticoagulant (LAC) profiles (dilute prothrombin time) showed borderline positivity. Other LAC tests (including Dilute Russell's Viper Venom Time and StaClot LA), rheumatoid factor, and anti-cyclic citrullinated peptide antibodies were all negative, which suggested a weak lupus anticoagulant. The patient also had protein electrophoresis done, which was negative for monoclonal spikes. Given the lack of infection, malignancy, and autoimmune disease, acute pericarditis with large pericardial effusion was attributed to his underlying progressive MRH. The patient was treated with three months of colchicine and a four-week tapering dose of indomethacin.

## Discussion

**Figure 6:**
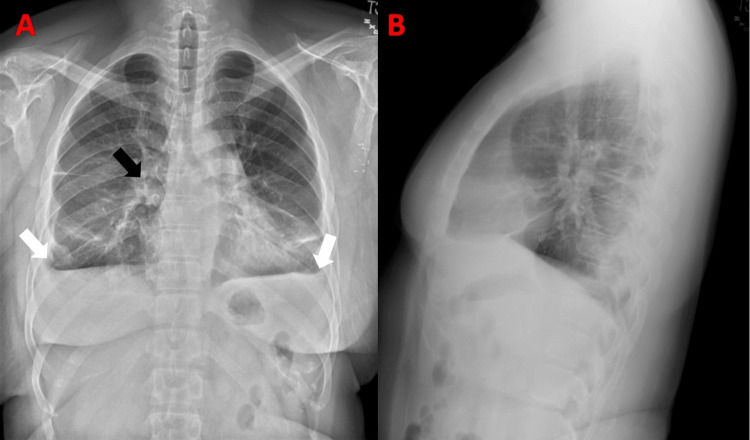
Chest x-ray posteroanterior (A) and lateral (B) views demonstrating patchy opacities over the hilar region (black arrow), haziness and blunting of costophrenic angle (white arrows) suggestive of pleural effusion. Possible cardiomegaly.

Reticulohistiocytosis comprises multicentric reticulohistiocytosis, generalized reticulohistiocytosis, and solitary reticulohistocytoma. Of all the three, MRH is the most common form of RH and is described in the literature as a class IIb non-Langerhans histiocytosis that frequently involves skin and joints [5]. Patients usually demonstrate symptoms and signs of symmetric polyarthritis and papulonodular cutaneous lesions (Figure 1). Pericardial involvement is a rare manifestation (Figures 5, 6), and around three cases have been reported in the literature so far [1,3,6]. Our case report is a valuable contribution to the literature, which aids clinicians in contemplating MRH as one of the differentials among patients presenting with pericardial effusion.

Diagnosis of MRH is based on the combination of clinical, histological, and laboratory findings. Patients typically have symmetric distal interphalangeal joint involvement along with shoulder, elbow, wrists, hip, knee, and feet arthritis. The bony erosions secondary to MRH are rapidly progressive, ultimately resulting in a condition called arthritis mutilans, observed in 45% of patients [7]. Skin nodules of MRH have a characteristic coral bead appearance and are more often observed around the ungual region and may also be observed on the mucosa, face, arms, and torso. Facial lesions were described as having a lion-like appearance in the literature [8]. Systemic MRH may present as pleural and pericardial effusion (Figures 5, 6), congestive heart failure, urogenital lesions, and mesenteric lymphadenopathy [5].

Systemic lupus erythematosus (SLE), Sjogren’s syndrome, rheumatoid arthritis (RA), tuberculosis, hypothyroidism, and diabetes are known to be associated with MRH. In addition, patients with solid or hematologic malignancies can present with MRH as the paraneoplastic syndrome. Such presentation is observed in 25-33% of MRH patients, and the symptoms usually abate with the management of the malignancies [7-9]. Due to the common features between MRH and other systemic autoimmune conditions, MRH is more prone to misdiagnosis. The most common misdiagnoses are rheumatoid arthritis and dermatomyositis. Differentiating features of MRH from RA include the rapid progression and early involvement of distal interphalangeal joints. Synovial biopsy aids in distinguishing RA and MRH further. While maculopapular nodules are characteristic of MRH, dermatomyositis manifests with heliotrope rash, Gottron papules, Gottron’s sign, and poikiloderma atrophicans vasculare and skin calcifications. Musculoskeletal involvement in dermatomyositis presents as proximal muscle weakness, unlike MRH, which involves arthritis.

Laboratory findings are nonspecific and include anemia, thrombocytosis, elevated erythrocyte sedimentation rate, and elevated C-reactive protein. These findings can also be confounded by the treatment with glucocorticoids. Synovial fluid analysis of the involved joints has been studied to be indicative of a non-inflammatory type of effusion in 75% of patients [10]. Around 20-75% of the patients with MRH have positive anti-nuclear, anti-citrullinated peptide, anti-Ro, and anti-SSA antibodies, which can explain the relation between MRH and connective tissue disorders [3,5]. Histologically, multinucleated giant cells and histiocytes are seen with eosinophilic ground glass cytoplasm. These cells stain positive for periodic acid-Schiff (PAS), CD163, HAM56, and CD68 and negative for Langerhans markers such as S-100, CD1a, CD19, and CD20 [7,8]. Recent studies have demonstrated positive Mac387, vimentin, CD43, CD45, and lysozyme in a few patients [8]. Imaging of MRH shows a characteristic absence of periarticular osteoporosis and periosteal osteoblastic activity, unlike inflammatory and juvenile idiopathic arthritis [4].

Early diagnosis is crucial to prevent disability and substantial disfigurement. Due to the low disease prevalence, there is no standard treatment protocol for MRH, and the current practice is based on the existing case reports. The similarity of clinical manifestations between MRH and rheumatologic conditions is proposed to be secondary to autoimmune or inflammatory pathology. Hence, the treatment with systemic glucocorticoids, methotrexate, bisphosphonates, and biologic agents are being employed with varied outcomes [11]. Prednisone can be started at a dose of 7.5 - ≤30 mg/day and then tapered to ≤7.5 mg/day based on the disease activity [12]. Chronic steroids are required in certain patients with frequent relapses. Non-steroidal anti-inflammatory drugs (NSAIDs) were previously used in the case of mild disease; however, due to the rapidly progressing nature of MRH, methotrexate is now commonly used once the initial diagnosis is made [5,12,13]. Methotrexate is shown to control joint and skin involvement in 28% and 38% of patients, respectively [5]. Leflunomide and azathioprine can be considered among patients with contraindications to methotrexate. Although hydroxychloroquine and sulfasalazine have not demonstrated clinical benefit in the past, the former may be used as a combination agent [12]. Cyclophosphamide provides complete resolution of joint and skin lesions in 20% and 27% of patients, respectively, and partial resolution in 40% and 45%, respectively [5]. Bisphosphonates can be the add-on agents in patients with poor disease control or to limit osteopenia/osteoporosis with chronic steroid use. However, their sole use is still debatable. Urine N-telopeptide correlates with bone turnover, and the levels gradually decrease with the bisphosphonates [12]. 

In patients with refractory disease to the aforementioned agents within four to six weeks, biologic agents such as tumor necrosis factor (TNF) and IL-6 inhibitors are studied. The response to these agents is based on the pathophysiology that the MRH is associated with increased release and expression of pro-inflammatory cytokines such as TNF, interleukin (IL)-6, IL-1b, and IL-8 [14]. Etanercept, adalimumab, and infliximab are the anti-TNF agents that are effective in MRH [15-17]. Tocilizumab is an IL-6 receptor antagonist considered as a treatment option [18]. Recently, anakinra, an IL-1 inhibitor, is studied to control the disease progression [5]. The JAK-inhibitors such as tofacitinib and upadacitinib are the alternative treatment options when steroids, disease-modifying antirheumatic drugs (DMARDs), and other biologic agents fail to improve the skin and joint lesions [5]. The superiority of one biologic agent over the other is difficult to interpret due to the low incidence of the disease.

## Conclusions

MRH is a rare non-Langerhans type histiocytosis that requires further studies on its management. The clinical picture often mimics autoimmune disorders like SLE and RA. Clinicians should have a high index suspicion of MRH for patients who present with arthritis symptoms. Its presentation as a pericardial effusion is infrequent, and our case report acts as an addition to the literature. This case report guides the clinicians to include MRH in the differential diagnosis of pericardial effusion and contributes to future retrospective studies.
